# A real-world observational study of dupilumab treatment in adult patients with prurigo nodularis^☆^

**DOI:** 10.1016/j.abd.2022.09.008

**Published:** 2023-03-23

**Authors:** Zhixin Zhang, Siyuan Li, Yang Wang, Jiahui Zhao

**Affiliations:** Department of Dermatology, Peking University First Hospital, Beijing Key Laboratory of Molecular Diagnosis on Dermatoses, National Clinical Research Center for Skin and Immune Disease, NMPA Key Laboratory for Quality Control and Evaluation of Cosmetics, Beijing, China

**Keywords:** Dupilumab, Prurigo, Therapeutics

## Abstract

**Background:**

Prurigo nodularis (PN) is a chronic inflammatory skin condition that has a significant unmet needs for effective treatment options.

**Objective:**

To assess the efficacy and safety of dupilumab in adult patients with PN refractory to traditional therapies.

**Methods:**

This ongoing, real-life study examined dupilumab treatment in 8 adult patients diagnosed with PN for at least 6 months. The included patients were prescribed 300 mg dupilumab biweekly for at least 16 weeks. Efficacy was the primary outcome by means of multiple standardized scale measurements while safety was also reported.

**Results:**

PN patients treated with dupilumab showed notable clinical improvement. After 16 weeks of dupilumab treatment, the mean Investigator Global Assessment (IGA) score reduced from 3.75 to 1.50. Patients mean Numerical Rating Scale Itch Intensity (NRSI), Dermatology Life Quality Index (DLQI), and Hospital Anxiety and Depression Scale (HADS) ratings reduced from 8.625, 15.13, and 14.50 to 1.563, 4.625, and 1.000 respectively. The majority of the patients (87.5%) reported dupilumab as effective while no adverse events have been reported.

**Study limitations:**

This study was limited to a small cohort of adult PN patients and a short-time observation period.

**Conclusions:**

Dupilumab is effective and tolerable in adult PN patients with significant improvement in skin lesions, itching, and quality of life.

## Introduction

Prurigo Nodularis (PN) is a chronic inflammatory skin condition characterized by the presence of multiple hyperkeratotic, pruritic nodules.^1^ Patients with PN often experience severe itch and thereafter, develop anxiousness about their conditions.^2^ Currently, the exact pathogenic mechanisms of PN remain unclear.^3^ Aside from its significant impact on patient’s quality of life, there are currently no FDA-approved treatments available, creating even more challenges to treat PN, resulting in a large unmet needs for patients whose symptoms are inadequately controlled with traditional therapies.^1^

Recent research suggests that T-helper 2 (Th2) cytokines, such as Interleukin-4 (IL-4) and Interleukin-13 (IL-13), may participate in PN pathogenesis.^4^ Herein, the authors examine the efficacy and safety of dupilumab, an Interleukin-4 Receptor-α (IL-4Rα) antagonist, as an off-label treatment for adult patients with refractory PN.

## Methods

This study is an ongoing observational study recruiting adult patients at the department of dermatology in a real-life setting in the hospital.

### Patients and design

This study consecutively enrolled patients who have been diagnosed with PN for at least 6 months and have failed to respond to traditional therapies. All patients included were evaluated by an accredited dermatologist with an IGA scale rating greater than or equal to 3, and an NRSI rating greater than or equal to 7. Patients who had received systemic immunosuppressants or phototherapy 3 months before the enrollment have been excluded from the study. Patients included in this study received subcutaneous injections of dupilumab following the standard dosage schedule (600 mg induction dose followed by 300 mg every other week) for at least 16 weeks. During the period of dupilumab treatment, patients were allowed to use accompanying topical glucocorticoids, oral antihistamines, topical antibiotic ointments, and traditional Chinese medicines as salvage treatments. Patient’s demographic information, medical history, and family history of allergic disorders have been reported at week 0 (baseline). Clinical response, blood test results, and adverse events (AEs) were closely monitored throughout the treatment period to aid in the evaluation of the efficacy of dupilumab therapy. Specifically, blood tests were performed at week 0 and week 16 of dupilumab treatment to assess white blood cell count (WBC), serum absolute eosinophil count (AEC), and total serum IgE (TIgE) levels. Additionally, patients’ Investigator's Global Assessment (IGA), Numerical Rating Scale Itch Intensity (NRSI), Dermatology Life Quality Index (DLQI), and Hospital Anxiety and Depression Scale (HADS) ratings were reported at week 0 and week 16 of dupilumab treatment. Written informed consent was obtained from all patients before enrollment. The study was conducted after gaining approval from the Clinical Research Ethics Committee of the Hospital.

### Skin specimen collection from PN patients

Human skin biopsy specimens were collected from 2 patients diagnosed with PN based on pruritic nodules on the lower extremities and 1 specimen of healthy control was obtained on the same site. The experiments on human samples were conducted after obtaining written informed consent and approval from the Clinical Research Ethics Committee of the hospital.

### Immunofluorescence

The primary antibody used for immunostaining was an Anti-interleukin-17A-receptor antibody (ab180904, Abcam) diluted at 1:50. This step was followed by a secondary antibody conjugated to FITC (ZF-0311, ZSGB-BIO) diluted at 1:250. Samples were examined with fluorescent microscopy (Leica).

### Statistics

Statistical analyses were performed with GraphPad PRISM (version 9.2.0) with Student’s *t*-test as indicated (*p < 0.05, **p < 0.001, ***p < 0.0005, ****p < 0.001, ns not significant). Numerical results were presented as mean ± Standard Error of the Mean (S.E.M.).

## Results

### Patients

Eight patients (62.5% female) diagnosed with moderate to severe PN for at least 6 months (mean duration 5.525 years [Standard Deviation: 6.6 years]) were included in the study. All patients have been previously treated with multimodal traditional therapies but failed to respond. Treatment with systemic immunosuppressants was discontinued in all included patients 3 months prior to the induction dose of dupilumab. No AEs were reported during the study period while no patient dropped out because of AEs due to dupilumab treatment.

Baseline demographics and disease characteristics are shown in Table 1. Three patients (37.5%) had a family history of allergic/atopic disorders. Three patients (37.5%) had a personal history of allergic/atopic disorders. More than half of the total population (62.5%) had comorbid diseases (Hepatitis B, 12.5%; diabetes, 25.0%; hypertension, 50.0%). In all patients (100%), the onset of PN was during their adults’ age (≥18 years old). Meanwhile, all of them (100%) have reported ineffective responses to their previous therapies prior to dupilumab injection (Table 1).

**Table 1:** Baseline demographics and disease characteristics of patients with PN (n = 8).

Variables	Patients	Age, yrs	Sex	History of allergic disorders	Family history of allergic disorders	History of PN, years	Previous therapies	Clinical response to previous therapies	Current therapies	Other concurrent complications (comorbidity)	Adverse events
	1	73	F	N/A	N/A	3	Topical glucocorticoids, oral antihistamines, topical antibiotic ointment, tripterygium glycosides, thalidomide, traditional Chinese medicine	Ineffective	Dupilumab 300 mg qow, topical glucocorticoids, topical antibiotic ointment	N/A	N/A
	2	74	F	Allergic rhinitis, chronic urticaria	Eczema, allergic rhinitis, asthma, chronic urticaria, prurigo nodularis	20	Topical glucocorticoids, traditional Chinese medicine	Ineffective	Dupilumab 300 mg qow, traditional Chinese medicine	N/A	N/A
	3	53	M	N/A	N/A	2	Topical glucocorticoids, oral antihistamines, topical antibiotic ointments, tripterygium glycosides	Ineffective	Dupilumab 300 mg qow, topical glucocorticoids	N/A	N/A
	4	48	M	Asthma, allergic rhinitis, chronic urticaria	Eczema, allergic rhinitis, asthma, chronic urticaria, prurigo nodularis	10	Topical glucocorticoids, traditional Chinese medicine	Ineffective	Dupilumab 300 mg qow, traditional Chinese medicine	Hepatitis B	N/A
	5	76	F	N/A	N/A	2	Topical glucocorticoids, oral antihistamines, traditional Chinese medicine	Ineffective	Dupilumab 300 mg qow, topical glucocorticoids, oral antihistamines, traditional Chinese medicine	Diabetes, hypertension	N/A
	6	58	M	N/A	N/A	5	Topical glucocorticoids, oral antihistamines, azathioprine, thalidomide, glycyrrhizin, gabapentin	Ineffective	Dupilumab 300 mg qow, topical glucocorticoids, oral antihistamines	Hypertension	N/A
	7	66	F	N/A	Allergic rhinitis	1.5	Topical glucocorticoids, topical antibiotic ointments, oral antihistamines, traditional Chinese medicine	Ineffective	Dupilumab 300 mg qow, topical glucocorticoids, oral antihistamines, traditional Chinese medicine	Hypertension	N/A
	8	64	F	Asthma, allergic rhinitis	N/A	0.7	Topical glucocorticoids, traditional Chinese medicine	Ineffective	Dupilumab 300 mg qow, topical glucocorticoids, oral antihistamines, traditional Chinese medicine	Diabetes, hypertension	N/A
**Value, n (%)**	8	Mean ± standard deviation: 64 ± 10.3	Male: 3 (37.5); Female: 5 (62.5)	3 (37.5)	3 (37.5)	Mean ± standard deviation: 5.525 ± 6.6	N/A	Effective: 0 (0); Ineffective: 8 (100)	N/A	Hepatitis B: 1 (12.5); Diabetes: 2 (25.0); Hypertension: 4 (50.0)	0 (0)

N/A- Not Applicable; n- number of subjects meeting criteria; PN- Prurigo nodularis; qow, Every 2 weeks.

### Efficacy of dupilumab treatment

**Figure 1:**
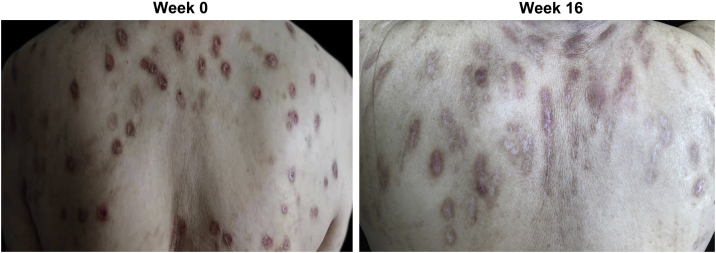
Clinical response of prurigo nodularis to dupilumab treatment. (A) Multiple reddish brown, firm, and hyperkeratotic skin nodules were distributed extensively across the upper back at week 0 (baseline) prior to dupilumab treatment. (B) Prurigo nodules showed significant regression accompanied by post-inflammatory hyperpigmentation and the absence of erosion or excoriation by week 16 of treatment with dupilumab.

At week 0, all enrolled patients suffered from widespread erythematous nodules; plaques distributed symmetrically on the extremities, hands, and trunk (Fig. 1A). After 16 weeks of dupilumab administration, skin lesions subsided in most cases (87.5%), indicated by reduced nodule size and number (Fig. 1B). One patient (12.5%) showed a poor response, with persistent itch and number of nodules. The number of nodules on his body was not significantly reduced while only rashes on the extensor side of the lower limbs were slightly decreased in size.

**Figure 2:**
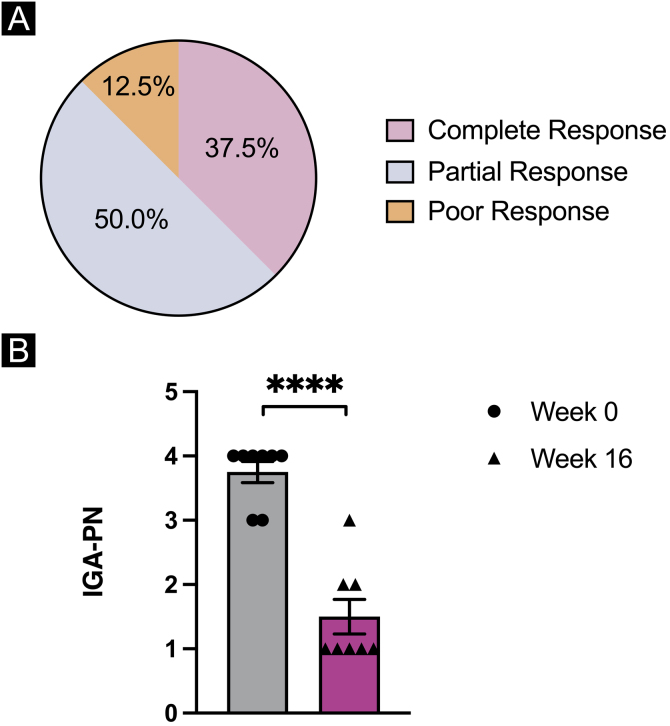
Statistical analysis of prurigo nodularis clinical parameters. (A) Overall response of dupilumab treatment in 8 patients with prurigo nodularis by week 16 of the treatment. (B) Investigator's Global Assessment (IGA) of the patients at week 0 and by week 16 of dupilumab treatment. Statistical significance was assessed by paired - tests, n = 8 patients; ****p < 0.001.

Overall, by week 16 of dupilumab treatment, two patients (25.0%) had a complete response of the treatment with a decrease in IGA for 3 grades; half of the population (50.0%) reported partial response to the treatment with a decrease in IGA for 2 grades; one patient (12.5%) reported poor response to dupilumab treatment with a less than 2 grades decrease in IGA (Fig. 2A). Dupilumab treatment led to a significant reduction in mean IGA scale ratings in the entire population of PN patients from week 0 to week 16 (p < 0.0001) (Fig. 2B) while no statistically significant differences were observed for laboratory results of WBC (p = 0.4137) and AEC (p = 0.2438) (Table 2). The mean IGA at week 0 (3.75) decreased to 1.50 by week 16 in the total population, while 5 patients (62.5%) had an IGA of 1 after 16 weeks of dupilumab treatment.

**Table 2:** The trend of WBC and AEC from week 0 to week 16 in 8 patients with prurigo nodularis treated with dupilumab.

	Patients' WBC (cells*10^9/L)		Patients' AEC (cells*10^9/L)	
	Week 0	Week 16	Week 0	Week 16
**Patient 1**	5.66	4.13	0.28	0.15
**Patient 2**	6.78	7.16	0.64	0.46
**Patient 3**	5.8	7.62	0.2	0.13
**Patient 4**	4.78	6.72	0.17	0.14
**Patient 5**	5.89	5.26	0.17	0.14
**Patient 6**	5.75	5.61	0.12	0.31
**Patient 7**	8.72	10.95	0.5	0.16
**Patient 8**	6.46	7.36	0.4	0.43

AEC- Serum absolute Eosinophil Count; WBC- White Blood Cell Count.

**Figure 3:**
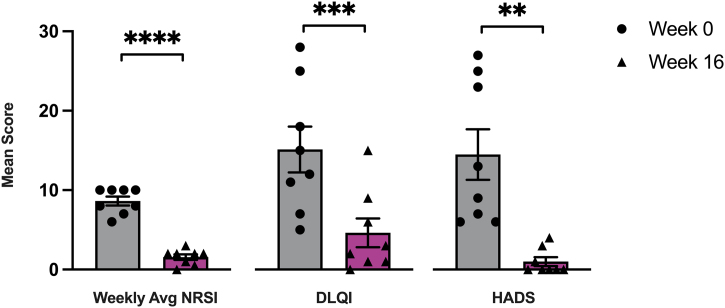
Statistical analysis of prurigo nodularis severity ratings. Patient-reported outcomes of weekly average Numerical Rating Scale Itch Intensity (NRSI), Dermatology Life Quality Index (DLQI), and Hospital Anxiety and Depression Scale (HADS) at week 0 and by week 16 of dupilumab treatment. Statistical significance was assessed by paired *t*-tests, n = 8 patients; **p < 0.001, ***p < 0.0005, ****p < 0.001.

Within 16 weeks, all patients reported subjective improvement in pruritus symptoms, quality of life, and anxiety levels. A statistically significant decrease was observed in weekly average NRSI ratings (p < 0.0001), DLQI ratings (p < 0.001), and HADS ratings (p < 0.01) by week 16 of dupilumab treatment (Fig. 3). The mean baseline (week 0) values of weekly average NRSI score (8.625), DLQI score (15.13), and HADS score (14.50) decreased to 1.563, 4.625, and 1.000 respectively, after 16 weeks of dupilumab treatment.

The majority of the patients (75.0%) have experienced a reduction in TIgE level during 16 weeks of dupilumab treatment (Table 3). Meanwhile, 4/8 patients showed significantly lower TIgE levels (mean % change = 52.58%) from week 0 to week 16 whereas the other patients exhibited no significant change.

**Table 3:** The trend of serum total IgE (kU/L) from baseline to week 16 in 8 patients with prurigo nodularis treated with dupilumab.

	Patients' serum total IgE (kU/L)	
	Week 0	Week 16
**Patient 1**	196.0	180.0
**Patient 2**	1093.0	711.0
**Patient 3**	1438.0	1592.0
**Patient 4**	5000.0	1175.0
**Patient 5**	72.8	68.8
**Patient 6**	18.5	42.0
**Patient 7**	515.0	268.0
**Patient 8**	273.0	134.0

kU/L- kilo Unit per Litre.

### PN lesional skin biopsy immunofluorescence staining

**Figure 4:**
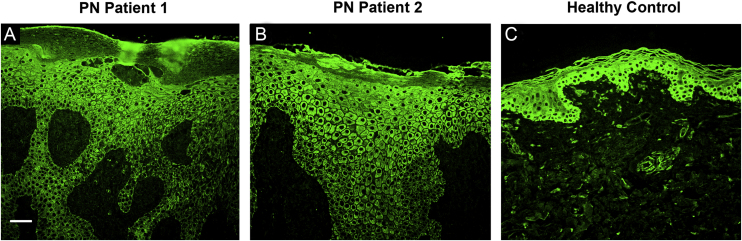
Immunofluorescence staining of IL-17RA (green) in two patients’ lesional skin biopsy specimens before dupilumab initiation. (A) Immunofluorescence staining of IL-17RA in PN Patient 1 showing hyperkeratinization, irregular acanthosis, and hypergranulosis. IL-17RA positive keratinocytes can be observed in parts of the stratum granulosum and the stratum spinosum. (B) Immunofluorescence staining of IL-17RA in PN Patient 2 showing hyperkeratinization, irregular acanthosis, and hypergranulosis. IL-17RA positive keratinocytes can be observed in large areas of the stratum granulosum and the stratum spinosum. (C) Immunofluorescence staining of IL-17RA in control specimen showing some IL-17RA positive keratinocytes in the stratum basale. Bar = 100 μm. IL-17RA- interleukin-17A receptor; PN- prurigo nodularis.

The immunofluorescence staining of two patients’ PN lesional skin specimens showed a large number of Interleukin-17A Receptor (IL-17RA) positive keratinocytes in the stratum granulosum and the stratum spinosum whereas the healthy control only has few IL-17RA positive keratinocytes in the stratum basale (Fig. 4).

## Discussion

PN is an inflammatory skin disease characterized by severe pruritus, the presence of multiple hyperkeratotic papules, nodular lesions, hyperpigmentation, and itch-associated anxiousness.^5^, ^6^ Due to a lack of FDA-approved treatment for PN, commonly prescribed therapies usually include topical and systemic use of antihistamines, steroids, gabapentinoids, immunosuppressants, antidepressants, and other neuromodulating agents that often put the patients at risk of severe side effects.^7^, ^8^ Therefore, many patients have a long-lasting, unsatisfied need for a safe and effective treatment for PN. In this real-life observational study, data on the efficacy and safety of dupilumab treatment in PN patients are presented. In adult patients with refractory PN who have failed to respond to traditional therapies, 16 weeks of dupilumab treatment resulted in statistically significant and clinically meaningful improvements in signs, symptoms, pruritus intensity, anxiety levels, depression levels, and quality of life with no cases of AEs been reported. In terms of the overall efficacy of dupilumab to PN, Calugareanu et al. have obtained an outcome of 50% of PN patients had a complete response, 41.7% of the patients had a partial response with 1/16 patients showed no response after 6 months of dupilumab treatment.^9^ We have reported similarly remarkable outcomes with 37.5% of PN patients had a complete response, 50% of PN patients had a partial response and 1/8 patient had a poor response to dupilumab. In the present study, symptom improvement after dupilumab initiation was rapid and significant. Improvements in skin lesions and nodules can be observed at early stages of 4 weeks of dupilumab treatment. By 16 weeks of dupilumab treatment, patients’ mean IGA reduced from 3.75 to 1.50. Similarly, Chiricozzi et al. has reported a reduction in IGA for at least 2 grades in 82.6% of the patients after 16 weeks of dupilumab treatment.^10^ These observations proved dupilumab’s tendency to elicit fast and notable responses of symptom reduction in PN patients. Additionally, patient-reported subjective improvements on pruritus intensity after dupilumab injection were even faster. In the cohort, some patients have reported a response to dupilumab in terms of pruritus reduction during week 2 of the treatment period. By week 16 of dupilumab treatment, a mean NRSI reduction of 7.063 was obtained from the present study. From previous studies, Zhai et al. observed a mean NRSI reduction of 7.89 by 2 weeks of dupilumab treatment.^11^ Beck et al. have reported pruritus improvement to nearly 0 in terms of NRSI for all 3 patients in 12 weeks of dupilumab treatment.^12^ These results confirmed dupilumab’s marked potency on pruritus relief in PN patients. Not only clinical symptoms have relieved significantly after dupilumab treatment, patients’ mental health and quality of life have also seen subjective improvements. Patients’ mean HADS has decreased from 14.50 at week 0 to 1.00 at week 16 of the treatment period showing significant improvements in mental health. Meanwhile, patients’ mean DLQI decreased from 15.13 to 4.625 after 16 weeks of dupilumab treatment. Giovannini et al. have reported a similar outcome of DLQI reduction from 16 to 1 after 12 weeks of dupilumab treatment in a patient with PN.^13^ The more notable and earlier reduction in DLQI from their reported case may be due to the age of their patient been adolescent whereas in this cohort were all adult patients. It is possible that dupilumab treatment is more effective in adolescent patients with PN. However, the authors still lack sufficient evidence due to the small number of studies publicly available. Dupilumab treatment has been proven safe in this cohort of adult PN patients as no AEs were reported. Its safety has also been proven by numbers of prior cases of dupilumab in PN with no AEs been detected.^10^, ^11^, ^12^, ^13^, ^14^, ^15^, ^16^ The present results suggest that dupilumab treatment is an effective and tolerable treatment option for adult patients with intractable PN.

Dupilumab is a fully human monoclonal antibody that acts as IL-4Rα antagonist to block the active biological effects of IL-4 and IL-13 in carrying out type 2 immune responses including some symptoms of PN.^4^, ^14^, ^15^, ^17^ Meanwhile, the exact pathogenesis of PN remains ambiguous. Some argued that PN is the long-term result of the chronic itch-scratch cycle of patients with chronic pruritus while others believe PN is a distinct disease that exists on its own.^18^, ^19^ Evidence suggests that PN may be a multi-factor disease arising from neurological, dermatological, and systemic origins with the possibility of undiscoverable causes in many cases.^20^ The specific mechanisms of dupilumab as an effective treatment for PN is not yet established. Perhaps the effectiveness of dupilumab in treating PN may be explained by disruption of the itch-scratch cycle via inhibition of neuronal IL-4Rα, which is required for transduction of chronic itch signaling, and results in attenuation of pruritus and skin inflammation.^21^ Experimental data showed that 37.5% of enrolled patients have a medical history of allergic disorders who had significantly decreased serum TIgE after dupilumab treatment. Meanwhile, a study conducted by Fukushi et al. observed the expression of IL-4 and IL-13 in PN lesional skin biopsies indicating the pathogenic role of Th2 cytokines in PN.^22^ These findings suggest that an excessive Th2 response could serve as a contributing factor in PN pathogenesis.

Recently, Belzberg et al. observed an increase in the expression of T Helper 17 (Th17)-associated genes (e.g., S100, LOR, K6, K17, etc.) in skin specimens of PN lesions.^23^ Therefore, we performed immunofluorescence staining of IL-17RA in 2 PN patients and a healthy control. The present result revealed a larger number of IL-17RA positive keratinocytes in samples of PN lesional skin when compared to the healthy control specimen. Based on this finding, the authors hypothesize that the observed upregulation in IL-17RA may be another underlying factor contributing to PN development. It is also possible that PN is clinically correlated to atopic dermatitis (AD) as Th17 related cytokines are found to be potential contributors in the pathogenesis of AD in recent years while dupilumab has a fair number of reported cases of being successful in treating patients with severe, intractable AD.^24^, ^25^, ^26^, ^27^, ^28^, ^29^, ^30^ These discoveries put forward the possibility of Th17/IL-17 inflammation-modulating therapies in becoming a beneficial treatment target for PN patients in the future. However, the specific pathogenic roles and signaling pathways of Th2 related cytokines, Th17 related cytokines, in AD and PN need further investigation with more disease models.

There have been a few clinical reports that have evaluated dupilumab response in PN patients before, but this study has its own strengths. First, the authors have used comprehensive, standardized measurement scales and surveys covering all aspects of the evaluation of disease symptoms, mental health, and quality of life in the cohort that resulted in detailed and accurate outcomes. Secondly, the present study was strictly constructed in real-life settings that in turn increased the practicability and generalizability of the application of dupilumab treatment in PN patients in everyday practice. However, this study also has some limitations. First, the sample size of this study was relatively small, with only 8 patients observed. Secondly, the sample was limited to adult PN patients, so pediatric outcomes are not clear. Thirdly, this study was restricted by the short 16 weeks treatment period, which means that long-term efficacy and safety evaluations are lacking.

## Conclusion

In conclusion, dupilumab treatment was proven effective in improving skin lesions, reducing itch, and ameliorating patients’ quality of life. Meanwhile, there were no AEs been detected, demonstrating dupilumab treatment as a safe and tolerable option for adult PN patients. Evidently, IL-4R was an important target in treating PN while other potential approaches targeting Th17 cytokines and their receptors may become future treatment options for patients with PN.

## IRB approval status

Reviewed and Approved by the Clinical Research Ethics Committee of the Peking University First Hospital, ID: 2021/223.

## Financial support

This work was supported by the National Natural Science Foundation of China (grant nº 81903213 to J. Zhao).

## Authors' contributions

Zhixin Zhang: Critical literature review; data collection, analysis and interpretation; preparation and writing of the manuscript; statistical analysis.

Siyuan Li: Critical literature review; data collection, analysis, and interpretation.

Yang Wang: Critical literature review; effective participation in research orientation; management of studied cases; manuscript critical review.

Jiahui Zhao: Critical literature review; data collection, analysis, and interpretation; effective participation in research orientation; management of studied cases; manuscript critical review; preparation and writing of the manuscript; study conception and planning.

## Conflicts of interest

None declared.
